# A Case of Sex Cord-Stromal Tumor Originating in the Retroperitoneal Space

**DOI:** 10.7759/cureus.29063

**Published:** 2022-09-11

**Authors:** Victor Șchiopu, Valentin Butnari, Vasile Țurcanu, Nicolae Ghidirim

**Affiliations:** 1 Department of Oncology, Nicolae Testemițanu State University of Medicine and Pharmacy, Chișinău, MDA; 2 Department of Surgical Gastroenterology, Oncological Institute, Chișinău, MDA; 3 Department of Surgery, Barking, Havering and Redbridge University Hospitals NHS Trust, London, GBR; 4 Department of Radiology, Nicolae Testemițanu State University of Medicine and Pharmacy, Chișinău, MDA

**Keywords:** sex cord-stromal tumor, retroperitoneal space, retroperitoneal tumor, retroperitoneal malignant lymphomas, surgical oncology, radiotherapy, chemotherapy

## Abstract

A 54-year-old man was seen in the clinic with the chief complaint of epigastric pain radiating to the left groin region and a predominant postprandial abdominal discomfort. Upon examination, a painless round mass with reduced mobility was felt in the left flank during deep palpation of the abdomen. His past medical history was irrelevant. Ultrasound and IV contrast-enhanced CT scan confirmed the presence of a large tumor and an exploratory laparotomy for removal of the tumor was performed. The microscopic examination of the specimen confirmed the primary diagnosis of retroperitoneal tumor (RPT) and identified it as an extragonadal germ cell tumor with a vestigial origin, which is a rare type affecting the kidney and adrenal gland. Primitive RPTs are histologically classified as mesenchymal and neuroectodermal or vestigial. These histological types are rarely found in surgical practice and are challenging to diagnose and treat due to the peculiarities of the site of origin where they develop. RPTs are extremely rare and approximately 80% are malignant and detected lately during the disease's course, commonly discovered in advanced stages of local or systemic evolution. Currently, surgical intervention remains the only effective method of treating these tumors.

## Introduction

Retroperitoneal tumors (RPTs) are a heterogeneous group of neoplasms originating in the retroperitoneal space (RPS) and do not refer to the retroperitoneal organs or the abdominal cavity. Malignant metastases from distant organs are also excluded from this group. Structurally, RPT can be solid and/or cystic. By histological origin, solid tumors can be divided into four groups: mesenchymal, neuronal, vestigial, and lymphoproliferative [1-3]. According to the literature, about 70-80% of primary retroperitoneal neoplasms are malignant, thus accounting for 0.1-0.2% of all malignant neoplasms [4]. Due to the low incidence of metastases (20-30%), the distant progress is more characteristic for vestigial and mesenchymal tumors, especially for leiomyosarcomas and neurogenic and angiogenic tumors (20-28%), and rarely for dedifferentiated liposarcoma (5.5%) [5].

Imaging investigations (CT and MRI) and biopsy play a crucial role in providing information necessary for differential diagnosis by determining the tumor characteristics, namely, the site, size, shape, its relationship to adjacent anatomical structures, their invasion, as well as the presence of metastases. CT scan can identify the tumor and its relationship with adjacent structures. It can also detect the presence of degeneration inside the tumor as well as calcifications, which are indirect signs indicating a malignant type and aggressiveness of the tumor process. Nuclear magnetic resonance might reveal the heterogeneity of the formation, as well as alternating cystic-degenerated areas with solid areas [6-8]. Pre-operative histopathological diagnosis based on biopsy together with other methods of diagnosis mentioned above are necessary to decide whether surgery is the first treatment choice, its timing among the other treatments, and its extension.

## Case presentation

**Figure 1:**
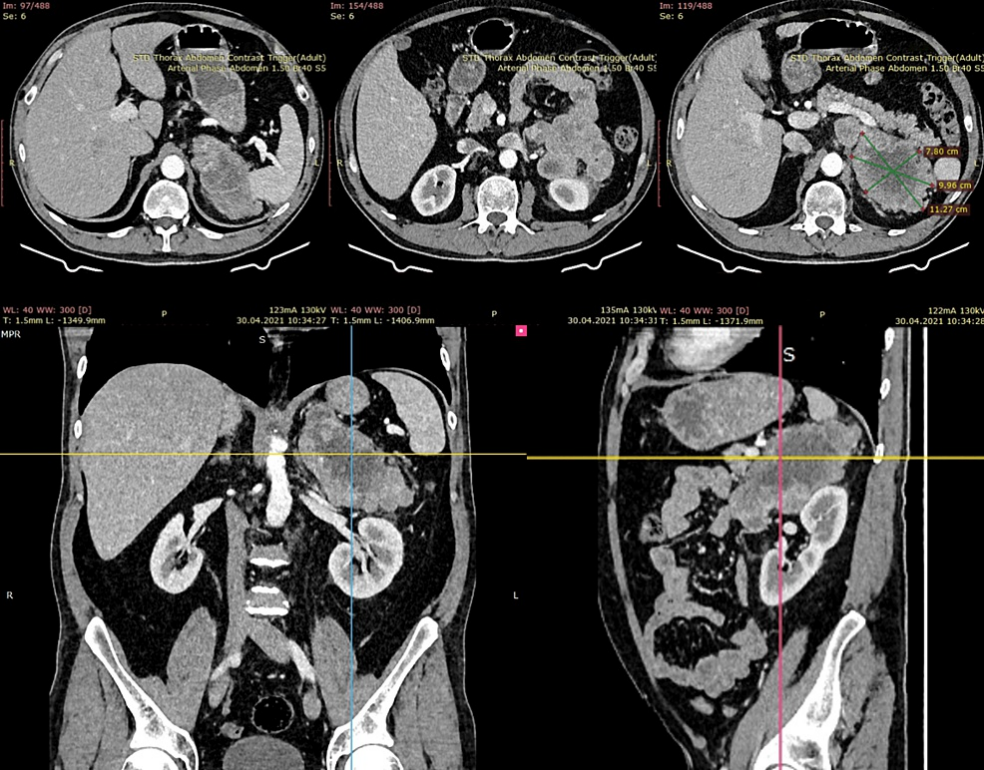
IV contrast-enhanced computed tomography (axial, coronal, and sagittal views) of the retroperitoneal lesion Enlarged formation of 11.6 x 9.0 x 10.3 cm, poorly circumscribed shape, mixed texture (solid-cystic), possibly originating from the small intestine. An expansive growth type, closely adhering to the spleen, upper pole of the left kidney, and left kidney hilum. The tail of the pancreas was not affected by this lesion.

A 54-year-old man, with no past surgical history, was referred to the doctor for epigastric pain radiating to the left groin region and a predominant postprandial abdominal discomfort. There was no associated history of weight loss, fever, or urinary complaints. The clinical examination focused on the abdomen revealed a distended abdomen and moderate pain in the left lumbar region where a hard round mass with reduced mobility extending superiorly into the left hypochondrium and inferiorly into the pelvis was felt. Signs of peritoneal irritation were negative. The liver and spleen were not palpable. Laboratory investigations including cancer antigen 125 (CA -125), carcinoembryonic antigen (CEA), alpha-fetoprotein (AFP), and beta-human chorionic gonadotropin (HCG) levels were within the normal range. Investigations performed were IV contrast-enhanced CT (Figure 1).

Due to the malignant appearance of this tumor on a CT scan, a pre-operative biopsy was considered a risk of dissemination [9,10]. The patient underwent median laparotomy and excision of the retroperitoneal mass. Upon inspection of the abdominal cavity, a hard palpable tumor was identified, proliferating from the RPS and displacing the spleen cranially and the splenic angle of the colon and the descending colon caudomedially. The splenic angle of the colon and the descending colon were mobilized via the Mattox maneuver, despite the technical problems occurring due to peritumoral adhesions. A big RPT approximately 20 cm in diameter was identified. The tumor partially invaded the upper pole and posterior surface of the left kidney and the lower pole of the spleen. We removed the tumor en bloc with the spleen, left adrenal gland, and left kidney. Total intraoperative blood loss was 2500 ml.

Gross characteristics

**Figure 2:**
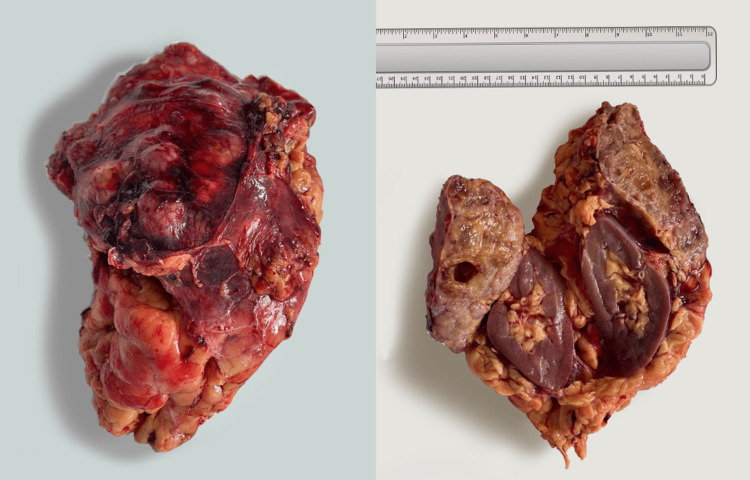
Macroscopic characteristics of the resected retroperitoneal tumor

The resected left kidney was 12 x 6 x 13 cm (Figure 2) with a resected tumor formation of 14 x 8 x 8.5 cm invading the renal vein and capsule. A tumor thrombus of 4.5 cm was determined on the vein segment. The tumor tissue was of grayish-pink color with heterogeneous texture with soft and bleeding areas; tissues similar to the adrenal gland were found in some areas in the foci. The peripheral tumor was surrounded by a yellowish adipose tissue of over 0.1 cm thick at the peripheral edges.

Microscopic characteristics

**Figure 3:**
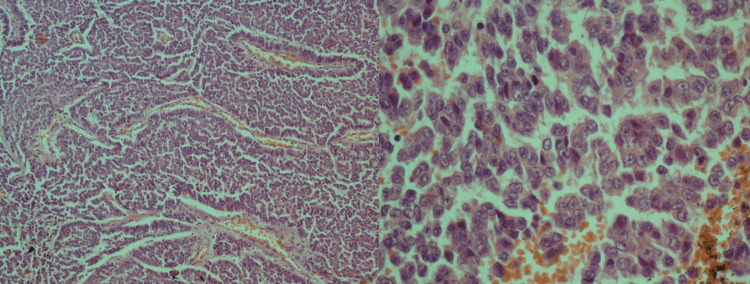
Adult granulosa cell tumor shows diffuse and trabecular growth; the tumor cells with coffee-bean nuclei, prominent nucleoli, and lack a “salt and pepper” appearance

The studied histological material was a proliferative tumor tissue of insular, trabecular, solid, and microfollicular architecture. Tumor tissue consisted of small and medium-sized polygonal cells with a low volume of cytoplasm and angled nuclei stamped in the form of coffee beans (Figure 3). Hypervascular stroma had a discrete inflammatory infiltration or myxomatosis. Lymphovascular invasion (LVi1) and negative perineural invasion (PN0) were present. The tumor invaded the adjacent adipose tissue. There was a tumor invasion in the renal capsule, and the kidney parenchyma was intact. The histological appearance of the spleen was normal without evidence of tumor invasion. The ureteral resection margins were negative. Immunohistochemical findings were as follows: tumor cells were positive for Melan A9 (+ diffuse), CD99 (+ diffuse), calretinin (+), and FOXL2 (+) and negative for pan-cytokeratin (PCK), epithelial membrane antigen (EMA), smooth muscle actin (SMA), desmin, neuron-specific enolase (NSE), S100, and GATA3.

The histopathological characteristics together with the immunohistochemical studies are specific for sex cord-stromal tumors (with granular cells of adult type) with all margins clear (R0-resection). The post-procedure hospital stay consisted of six days without any complications. Following a 3.5-month postoperative period, the patient showed no clinical signs of recurrence or distant progress. A routine imaging (MRI) examination was carried out over six months after surgery.

## Discussion

The RPS is a virtual anatomical space. It is bounded anteriorly by the parietal peritoneum, posteriorly by the intraperitoneal fascia, upward by the diaphragm, and laterally by the fold of the peritoneum from the lateral abdominal wall to the posterior abdominal wall and extending downward to promontory and linea terminalis or pelvic brim. The RPS content comprises adipose tissue, layered by fascia, which includes the retroperitoneal organs, blood vessels, and peripheral nerves. The clinical signs of tumors that invade this space are nonspecific and often secondary, depending on the tumor topography and relationship with the adjacent anatomical structures, found at the time of diagnosis. Smaller tumors (less than 5 cm) are usually “clinically silent” and are discovered accidentally the majority of the time [11,12]. As the tumors grow (over 10 cm), symptoms appear due to compression or invasion of the adjacent organs [13-17].

Clinical manifestations are not specific to this histological type of tumor. Most RPTs are of mesodermal origin, the most common being liposarcoma, leiomyosarcoma, and undifferentiated pleomorphic sarcoma. Although germ cell tumors (GCTs) are often present in the testes or ovaries, these types of extragonadal tumors are rare. These tumors arise from the remnants of primordial germ cells that accidentally remain within the aforementioned spaces during their physiological migration in the urogenital ridge, a process that occurs between the second and the eighth week of embryonic development. Tumors originating from extragonadal germ cells are often detected near the midline, especially between the T6 and S2 vertebrae; whereas the RPS is the second most common site of their development after the mediastinum. The imagistic studies show that tumors developed from extragonadal germ cells have characteristics similar to a gonadal tumor.

GCTs can occur at any age and can be of the adult (95%) or juvenile (5%) type, with subtypes distinguished by clinical and histological features [18]. The clinical behavior specifically for sex cord-stromal tumors is poorly understood and the overall experience rests on less than 50 case reports published. There are no evidence-based guidelines available to guide clinicians in the management and long-term surveillance of these kinds of pathology.

## Conclusions

The extragonadal GCTs are aggressive neoplasms that may occur almost anywhere; however, the most common sites are the mediastinum, retroperitoneum, and pineal gland. High-precision imaging investigations, such as CT and nuclear MRI, are still the main imaging methods for diagnosing RPT and planning surgical treatment. The rarity of these tumors and the complexity of their treatment require multidisciplinary management in specialized centers to improve oncologic and clinical outcomes.
